# T follicular helper cell responses to SARS‐CoV‐2 vaccination among healthy and immunocompromised adults

**DOI:** 10.1111/imcb.12635

**Published:** 2023-03-23

**Authors:** Mollie Ailie Acheson Boyd, Alexandra Carey Hoppe, Anthony D Kelleher, C Mee Ling Munier

**Affiliations:** ^1^ Immunovirology and Pathogenesis Program The Kirby Institute UNSW Sydney NSW 2052 Australia; ^2^ St Vincent's Hospital Sydney NSW 2010 Australia

**Keywords:** CD4 T cells, circulating T follicular helper cells (cTfh), fine needle aspiration (FNA), fine‐needle biopsy (FNB), Lymph node, SARS‐CoV‐2 vaccination, T follicular helper cells (Tfh)

## Abstract

The worldwide rollout of severe acute respiratory syndrome coronavirus 2 (SARS‐CoV‐2) vaccinations in the last 2 years has produced a multitude of studies investigating T‐cell responses in the peripheral blood and a limited number in secondary lymphoid tissues. As a key component to an effective immune response, vaccine‐specific T follicular helper (Tfh) cells are localized in the draining lymph node (LN) and assist in the selection of highly specific B‐cell clones for the production of neutralizing antibodies. While these cells have been noted in the blood as circulating Tfh (cTfh) cells, they are not often taken into consideration when examining effective CD4^+^ T‐cell responses, particularly in immunocompromised groups. Furthermore, site‐specific analyses in locations such as the LN have recently become an attractive area of investigation. This is mainly a result of improved sampling methods *via* ultrasound‐guided fine‐needle biopsy (FNB)/fine‐needle aspiration (FNA), which are less invasive than LN excision and able to be performed longitudinally. While these studies have been undertaken in healthy individuals, data from immunocompromised groups are lacking. This review will focus on both Tfh and cTfh responses after SARS‐CoV‐2 vaccination in healthy and immunocompromised individuals. This area of investigation could identify key characteristics of a successful LN response required for the prevention of infection and viral clearance. This furthermore may highlight responses that could be fine‐tuned to improve vaccine efficacy within immunocompromised groups that are at a risk of more severe disease.

## Graphical abstract

T follicular helper cells (Tfh) are a key component of an effective immune response, with improved sampling *via* ultrasound‐guided fine‐needle biopsy/fine‐needle aspiration allowing for noninvasive retrieval of these cells. This review focuses on both the Tfh and peripheral blood circulating Tfh responses after SARS‐CoV‐2 (severe acute respiratory syndrome coronavirus 2) vaccination in both healthy and immunocompromised individuals.

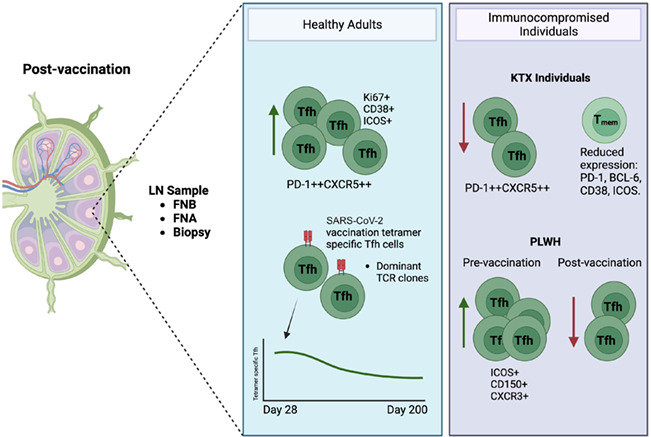

## INTRODUCTION

The effort of vaccination and its global application is one of the best ways to reduce the burden of infectious disease. Implemented first to eliminate the spread of smallpox, regular vaccination for diseases such as polio and measles has led to their elimination in developed countries, significantly reducing morbidity and mortality (particularly in children).^1^ More recently, the severe acute respiratory syndrome coronavirus 2 (SARS‐CoV‐2) vaccination has played a substantial role in significantly reducing the severe consequences of this infection. This is particularly important for vulnerable groups such as the immune compromised, who are at most risk of complications from disease.^2^, ^3^

While this group receives a substantial benefit, some individuals are unable to mount an appropriate response to vaccination.^4^, ^5^, ^6^ Those undergoing immunosuppressive treatment (such as monoclonal antibody therapy, alkylating agents or organ transplant recipients) and patients with cancer often have issues with seroconversion.^4^, ^7^ In the case of the SARS‐CoV‐2 vaccinations, these communities often developed lower antibody levels after vaccination and, in some studies, lower levels of vaccine‐specific T cells.^6^, ^8^

T follicular helper (Tfh) cells are a specialized CD4^+^ T‐cell subset found in secondary lymphoid organs such as the lymph node (LN). They play a vital role in B‐cell survival, clonal expansion, somatic hypermutation and affinity maturation, leading to highly specific and class‐switched antibodies.^9^ While responses to vaccination occur in the LN, access to this environment for analysis of the human immune response is difficult and can be invasive. Circulating Tfh cells (cTfh) have been used as a surrogate for LN Tfh cells and are easily measured in the peripheral blood.^10^ This subset has been shown to correlate with neutralizing antibody (NAb) levels following various vaccinations, which are key for the prevention of infection and clearance of acute viral infections.^10^, ^11^, ^12^

In this review, we focus on Tfh cells in response to SARS‐CoV‐2 vaccinations in healthy and immunocompromised individuals in both the site‐specific LN and peripheral blood. This comparison could assist in understanding the differences between these groups, which may be fine‐tuned to improve vaccine efficacy within immunocompromised individuals that are at a risk of more severe disease.

## T FOLLICULAR HELPER CELLS

In the T‐cell zone of the LN, naïve CD4^+^ T cells are primed by antigen‐presenting dendritic cells *via* peptide engagement of the cognate T‐cell receptor (TCR), leading to the downregulation of C–C chemokine receptor type 7 (CCR7).^13^ The presence of particular cytokines, such as interleukin‐6 and interleukin‐21 in the LN microenvironment at the time of priming, lead these cells toward a Tfh differentiation pathway, hallmarked by the upregulation of C–X–C chemokine receptor type 5 (CXCR5) on the cell surface.^14^ Allowing for the migration of these CD4^+^ T cells from the T‐cell zone to the T–B‐cell border, where they express the transcription factor B‐cell lymphoma 6 (BCL‐6) and the key molecule inducible T‐cell costimulator (ICOS)^15^, ^16^ it is in this location they are termed pre‐Tfh cells.^17^ Upon interaction with activated B cells presenting the cognate antigen and critical signaling through molecules such as ICOS/ICOS ligand, CD40 ligand/CD40 and CD28/CD86, the pre‐Tfh cells undergo differentiation into Tfh cells and are able to migrate into the B‐cell follicle to provide B‐cell help and underpin the formation of germinal centres.^18^, ^19^, ^20^, ^21^ It is at this location that the activated Tfh cells engage with B cells to support their expansion and increase antibody specificity.^19^, ^22^ It is believed that some of these germinal center Tfh cells migrate out of the LN and can be found in the peripheral blood as cTfh cells.^10^, ^23^, ^24^ The source of cTfh is, however, not entirely clear. An alternative proposal by He *et al*. demonstrated *via* murine model that cTfh cells were generated from pre‐Tfh cells prior to their migration to the germinal center of the LN.^22^

**Figure 1:**
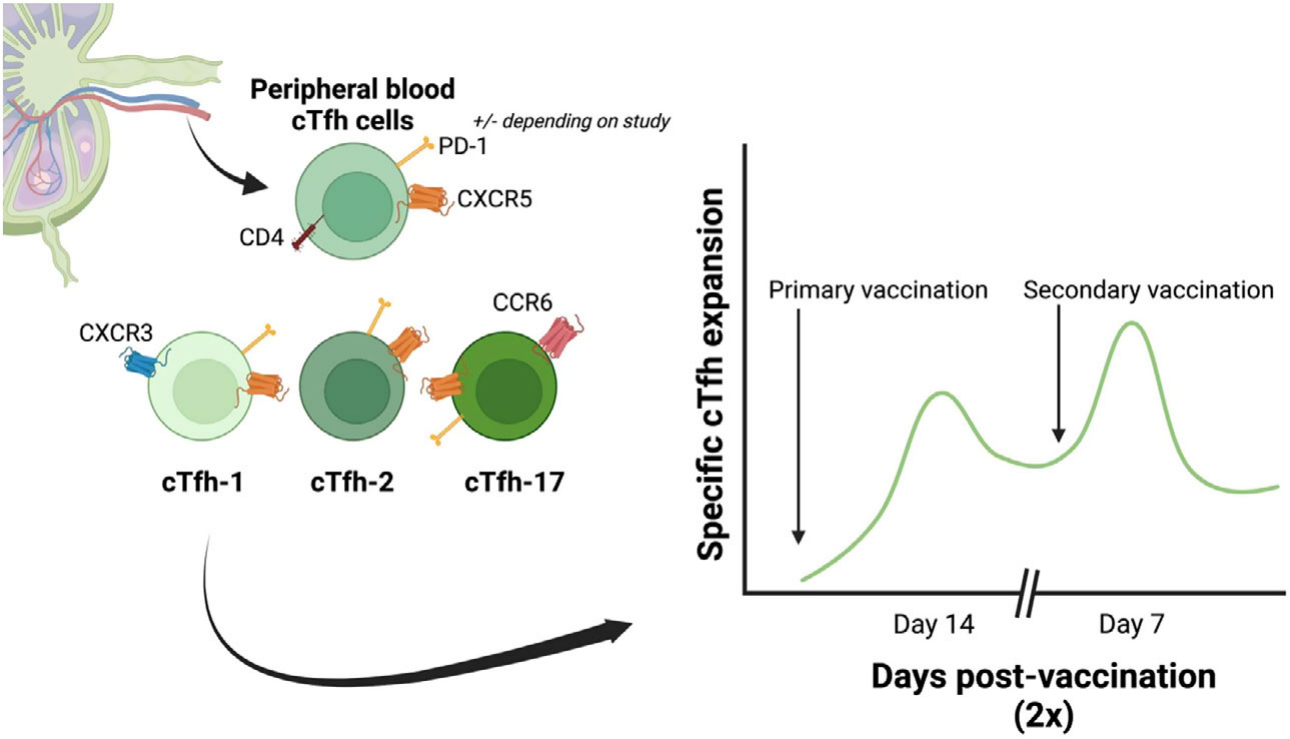
Dynamics of peripheral blood circulating T follicular helper (cTfh) cells after vaccination. In healthy individuals, cTfh type 1 (PD‐1^+^ CXCR5^+^CXCR3^+^) cells increase at day 14 of the primary immune response and at day 7 in the secondary response (in yellow fever and influenza vaccination). This cell type has been noted to correlate with higher levels of neutralizing antibodies after vaccination. This figure was created with BioRender. CCR6, C–C chemokine receptor type 6; CXCR3, C–X–C chemokine receptor type 3; CXCR5, C–X–C chemokine receptor type 5; PD‐1, programmed cell death protein 1.

cTfh cells have been shown to express similar phenotypic markers as *bone fide* LN Tfh cells, such as CXCR5, programmed cell death protein 1 (PD‐1) and often ICOS; however, they lack BCL‐6 lymphoma 6 expression.^12^, ^25^ Defined by their expression of CXCR3 and CCR6, cTfh can be further subtyped into cTfh‐1, cTfh‐2 and cTfh‐17 (Figure 1). These cTfh subsets have been detected in response to several vaccinations and infections (summarized in Table 1).^26^, ^27^, ^28^ Similar subtyping has been performed in macaque LNs with the separation of Tfh cells into Tfh‐1, Tfh‐2 and Tfh‐17, as has been performed in human blood previously (Table 1).^29^ CXCR3^+^ Tfh cells have also been observed in the human tonsil but were not defined as Tfh‐1 in this study.^23^ This subtyping could assist in understanding Tfh migration within and from the LN, and their relationship to peripheral blood cTfh cells.^29^, ^30^

**Table 1:** Summary of immunophenotyping markers to identify T follicular (Tfh) and circulating T follicular helper (cTfh) cells in the immune response to vaccination and infection.

Cell type	Location	Identifying markers (including CD3^+^CD4^+^)	Immune response to vaccination and infection
Tfh	Lymph node	CD45RA^−^CD25^−^CD127^+(^ ^a^ ^)^, PD‐1^++^ CXCR5^++^ BCL‐6^+^ and ICOS^+^ are also used^55^, ^56^, ^57^	These cells undergo expansion after SARS‐CoV‐2 and influenza vaccination and persist 200 days after inoculation.^55^, ^57^
Tfh‐1	Lymph node/Tonsil	PD‐1^++^ CXCR5^++^ CXCR3^+^ ^29^	Accumulate in the LN of SIV‐infected macaques and produce IFN‐γ.^29^, ^30^ CXCR3^+^ Tfh cells have been identified in the tonsil to have shared TCR clonality with cTfh1 and are increased in HIV^+^ inguinal LNs^23^, ^66^
Tfh‐2	Lymph node	PD‐1^++^ CXCR5^++^ CCR4^+^ ^30^	Have been observed in the LN of macaques.^30^
Tfh‐17	Lymph node	PD‐1^++^ CXCR5^++^ CCR6^+^ ^30^	Depleted in the LN of SIV‐infected macaques.^30^
cTfh	Peripheral blood	CD45RA^−^CD25^−^CD127^−(^ ^a^ ^)^, PD‐1^+^CXCR5^+55^; alternatively, CD45RA^−^ PD‐1^+/−^CXCR5^+^ ^23^, ^25^, ^26^	Counterpart to Tfh cells in the peripheral blood, potential correlate of vaccine response and biomarker for disease.^11^, ^23^, ^27^
cTfh‐1	Peripheral blood	PD‐1^+^CXCR5^+^CXCR3^+^ ^26^	Dominant phenotype in yellow fever and influenza vaccination–specific cTfh cells.^10^, ^25^ Positive correlation with neutralizing antibody levels.^10^, ^42^
cTfh‐2	Peripheral blood	PD‐1^+^CXCR5^+^CXCR3^−^CCR6^−^ ^28^	Often observed in the blood following parasitic infections and not often found after vaccination.^28^
cTfh‐17	Peripheral blood	PD‐1^+^CXCR5^+^CXCR3^−^CCR6^+^ ^26^, ^28^	Higher levels of these cells have been correlated with lower neutralizing antibodies upon yellow fever vaccination.^10^

^a^ CD45RA^−^ used to identify memory CD4 and CD25^−^CD127^+^ cells can be used to exclude regulatory T cells, not consistently used by all studies to define Tfh cells.

CXCR3, C–X–C chemokine receptor type 3; CXCR5, C–X–C chemokine receptor type 5; CCR4, C–C chemokine receptor type 4; CCR6, C–C chemokine receptor type 6; ICOS, inducible T‐cell costimulator; IFN, interferon; LN, lymph node; PD‐1, programmed cell death protein 1; SARS‐CoV‐2, severe acute respiratory syndrome coronavirus 2; SIV, simian immunodeficiency virus; TCR, T‐cell receptor.

## RESPONSES TO SARS‐COV‐2 VACCINATION AMONG HEALTHY ADULTS

SARS‐CoV‐2 emerged in late 2019 and quickly spread across the globe, causing approximately 674 million infections and over 6.8 million deaths at the time of writing.^31^ Less than a year later, multiple SARS‐CoV‐2 vaccinations were developed and rolled out, resulting in substantially reduced morbidity and mortality of infection.^32^, ^33^, ^34^, ^35^, ^36^, ^37^

### Peripheral blood responses in healthy individuals

Analyzing the expansion of antigen‐specific cTfh cells in the blood has proven valuable for understanding the immune response to yellow fever vaccination (YFV) and hepatitis B vaccination, with these responses positively correlated with NAb levels.^10^, ^11^ In a secondary response to influenza vaccination, cTfh cells peaked 7 days after vaccination compared with 14 days after vaccination in a primary response to YFV and SARS‐CoV‐2 vaccination.^10^, ^12^, ^25^, ^38^, ^39^ Antigen‐specific cTfh cells were found to correlate with spike immunoglobulin G antibody levels in infection‐naïve healthy individuals 10–12 days following the second SARS‐CoV‐2 messenger RNA (mRNA) vaccination.^40^ Increased frequencies of ICOS^+^ cTfh‐1 and ICOS^+^CD38^+^ cTfh cells have also been correlated with higher immunoglobulin G antibody levels in separate studies of influenza and pneumococcal vaccination.^41^, ^42^, ^43^ Interestingly, these cells have been shown to display increased TCR clonality, indicating their probable antigen specificity compared to their ICOS^‐^CD38^‐^cTfh counterparts, emphasizing the importance of these markers in the immune response to vaccination.^44^

To detect SARS‐CoV‐2 spike–specific CD4^+^ T cells in the blood, Wragg *et al*.^45^ used an HLA‐DRB1*15:01‐restricted tetramer (S_751–767_).^45^ Following initial SARS‐CoV‐2 vaccination [*n* = 7 BNT162b2 (Pfizer), *n* = 2 ChAdOx nCov‐19 (AstraZeneca/Oxford), *n* = 1 NVX‐Cov2372 (Novavax)], expansions of tetramer‐specific cTfh cells were identified in SARS‐CoV‐2 infection‐naïve individuals but upon secondary vaccination the same level of expansion was not observed.^45^ Interestingly, however, Painter *et al*.^46^ identified an expansion of SARS‐CoV‐2–specific cTfh cells (following peptide pool stimulation) 1 week after secondary vaccination in 36 individuals that received the mRNA SARS‐CoV‐2 vaccination (94% received BNT162b2 and 6% mRNA‐1273).^46^ The differences in results could be a result of several factors such as the flow cytometry gating, both studies defined cTfh as CXCR5^+^ memory CD4^+^ T cells, with Painter *et al*. excluding the CD45RA^+^CD27^+^CCR7^+^‐naïve T‐cell population and Wragg *et al*. excluding the CD45RA^+^CCR7^+^ population. Further differences could be a result of smaller cohort size in the latter study and the detection of antigen‐specific cTfh cells *via* tetramer, compared with peptide pool stimulation, as the true magnitude of these cells could be underestimated because of their restriction to a particular SARS‐CoV‐2 TCR epitope.

An interesting observation was made by Zhang *et al*.^38^ who studied cTfh cells in response to four different coronavirus disease 2019 (COVID‐19) vaccines [mRNA‐1273 (Moderna), BNT162b2 (Pfizer), Ad26.COV2.S (Janssen) and NVX‐Cov2372 (Novavax)].^38^ A SARS‐CoV‐2 spike peptide pool was used to expand and detect antigen‐specific cTfh cells and showed that the percentage of these cells was significantly lower following the Janssen vaccination compared with the other vaccines tested at all timepoints. A nonsignificant difference was also observed between the median percentage of cTfh cells between the two mRNA vaccines. Principal component analysis of cellular and neutralization data revealed that while the Moderna and Pfizer vaccines generate similar cellular responses, the latter tends to have more “heterogeneity” in both the CD4^+^ and CD8^+^ T cells over time, with regard to intracellular cytokine expression and activation. This flags potential sources of variability when investigating in‐depth COVID‐19 vaccine cellular immune responses, even between the two mRNA‐based vaccines, and should be taken into consideration in future studies.

The aforesaid studies highlight the different approaches used to identify SARS‐CoV‐2 spike–specific cTfh following vaccination. Tetramer‐based studies are performed *ex vivo* and hence allow further phenotypic characterization of the spike‐specific cTfh; markers for activation and chemokine receptors such as CXCR3 and CCR6 may be included with tetramer staining. Peptide stimulation–based assays can be performed *in vitro* without HLA‐typing and hence can include larger numbers of vaccinees. This methodology allows the assessment of a broader SARS‐CoV‐2 spike–specific cTfh response; however, further phenotypic characterization maybe limited because of the changes in certain markers during antigen stimulation.

By incorporating the data from both tetramer and peptide stimulation–based assays, a more comprehensive picture of how SARS‐CoV‐2 vaccines elicit cTfh responses can be gleaned. The subdivision of antigen‐specific cTfh into cTfh‐1, cTfh‐2 and cTfh‐17 (Table 1) provides further characterization of the cTfh vaccine response. Previous vaccination studies have found that antigen‐specific cTfh‐1 were positively correlated with NAbs following YFV.^10^ In that same study, the antigen‐specific cTfh‐1 were detected increasingly until 14 days following YFV and in other studies, it has been seen 7 days following influenza and human papillomavirus vaccinations.^10^, ^25^, ^47^

This cTfh‐1 skewing of antigen‐specific cTfh was also observed by Wragg *et al*.^45^ following the second dose of SARS‐CoV‐2 vaccination.^45^ This was similar in a study that measured cTfh numbers *via* cells/μL, in which the authors found a significant increase in the cTfh‐1 counts per μL of blood, but was limited to the first dose of the mRNA‐1273 vaccination.^48^ There was a twofold increase also noted by Samanovic *et al*. after the first dose of BNT162b2 vaccination (and a slight increase upon second dose) among the cTfh cells (PD‐1^+^CXCR5^+^) that expressed ICOS^+^CD38^+^, wherein these activated cells have been correlated with antibody responses and overlapping clonality after successive influenza vaccinations.^49^, ^50^ The polarization of the cTfh cells seems to occur after multiple different vaccinations, including the SARS‐CoV‐2 mRNA platform, and at times correlates with increased protection in the form of spike‐immunoglobulin G and neutralizing antibodies.^50^

cTfh‐1 polarization, however, has not been found ubiquitously across all cTfh SARS‐CoV‐2 vaccination responses. Following the third dose of CoronaVac, antigen‐specific cTfh‐17 expansion dominated the total spike‐specific cTfh cell response at 2‐ and 8‐weeks after vaccination.^51^ It is unclear whether this was observed following the first or second CoronaVac vaccinations, as this study only investigated these cTfh subtypes after the third dose. cTfh‐1 and cTfh‐17 cells have further been investigated after two doses of the NVX‐CoV2373 (Novavax) vaccination, with higher spike‐specific cTfh‐17 cells seen at day 7 after the first dose that slightly correlated (*r* = 0.35, *P* = 0.044) with spike immunoglobulin G titers 14 days later (day 21).^52^

A recent study concluded that cTfh‐17 cells are able to maintain better immune memory than the cTfh‐1 and cTfh‐2 subsets, as they made up the majority of the antigen‐specific cTfh responses 1‐month after hepatitis B and influenza vaccinations.^53^ However, in the YFV study by Huber *et al*.^10^ this subset was inversely correlated with NAb levels.^10^ Clearly, there is still more to learn about the dynamics of the antigen‐specific cTfh cells responding to the different vaccine platforms and how these cells shape the immune response to SARS‐CoV‐2 vaccination.

### Lymph node responses in healthy adults

Following deltoid intramuscular vaccination, establishment of the immune response occurs in the axillary draining LN. While there is an abundance of research on vaccine responses in peripheral blood, human LN studies are limited. Investigating this crucial location would allow for more in‐depth characterization of the cellular response to vaccination in both healthy and immunocompromised groups.

It is only in recent years that ultrasound‐guided fine‐needle biopsies (FNBs;sampling *via* capillary action) or fine‐needle aspirates (FNA; sampling *via* aspiration) have been used to collect human LN cells during infection (such as HIV) or following vaccination.^54^, ^55^, ^56^ In 2020, Turner *et al*.^54^ applied this technique to study LN responses following influenza vaccination, in which their main focus was to investigate the complexities and clonality of germinal center B cells and plasmablasts.^54^ While there was no focus on Tfh or CD4^+^ T cells in general, this was one of the first papers to use this sample type after vaccination in humans.

A comprehensive analysis of CD4^+^ Tfh cells was recently reported by our group in which we performed FNBs of the draining and contralateral axillary LNs pre‐ and 5 days after seasonal influenza vaccination.^55^ An increase in the absolute number of GC‐Tfh cells was observed exclusively in the draining LN after vaccination compared with before vaccination. These cells were highly activated and had recently proliferated, as measured by the coexpression of CD38, ICOS and Ki‐67, respectively. Prior to the COVID‐19 pandemic and the subsequent delivery of multiple SARS‐CoV‐2 vaccinations, there were very few studies investigating the LN through FNB/FNA following vaccination.

**Figure 2:**
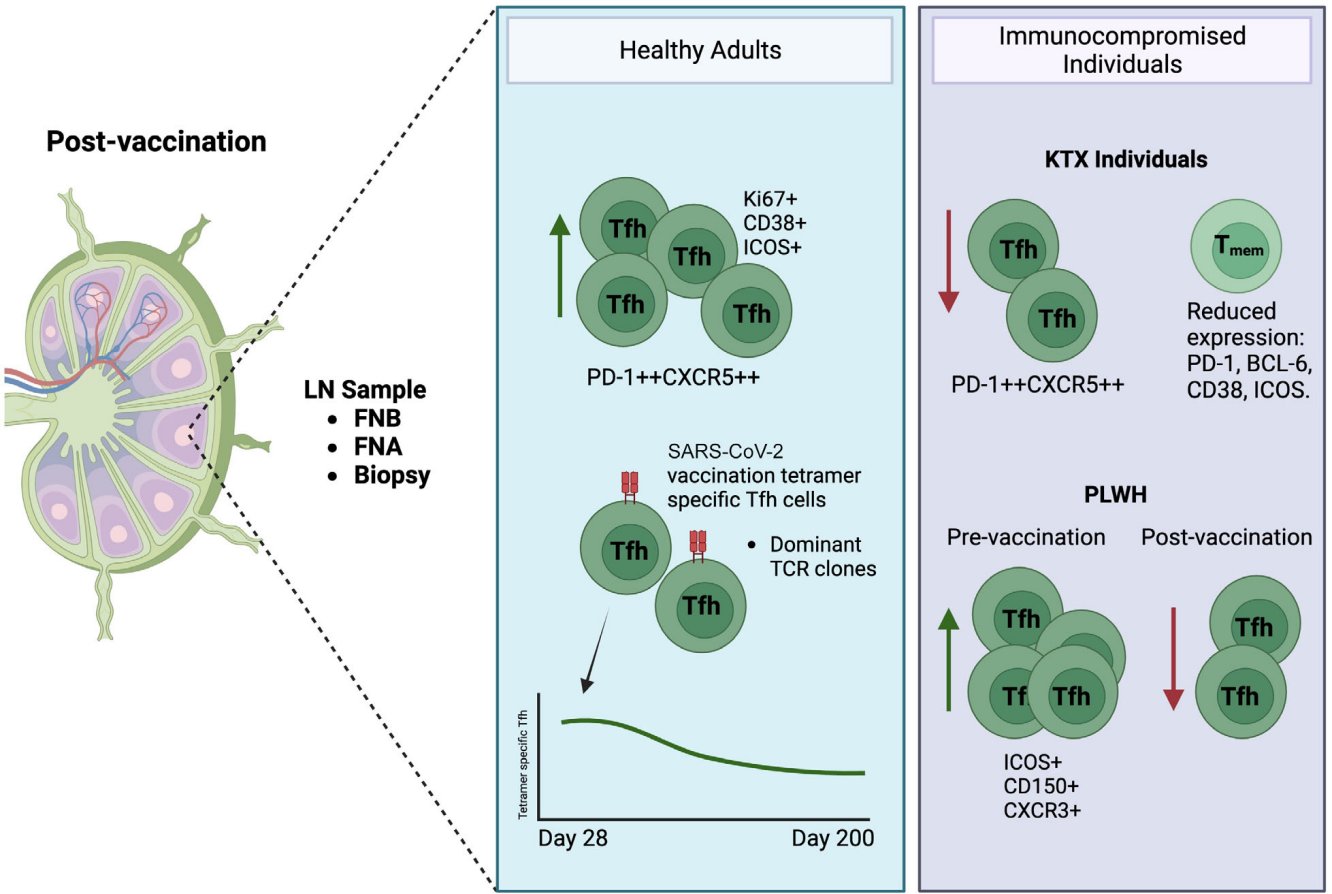
T follicular helper (Tfh) vaccination responses in lymph node in healthy and immunocompromised individuals. A range of different sampling methods of axillary and inguinal lymph nodes following influenza and severe acute respiratory syndrome coronavirus 2 (SARS‐CoV‐2) vaccination in healthy individuals have revealed a large increase in Tfh cells that are proliferating and highly activated. Tfh cells that are specific to antigens presented by messenger RNA (mRNA) vaccination in particular have considerable longevity in this site. In immunocompromised individuals after vaccination, these cells are reduced in number but also lack the expression of key markers for activation, migration and B‐cell interaction. The tetramer‐specific Tfh graph is adapted from Mudd *et al*.^57^ The image was created with BioRender. CXCR3, C–X–C chemokine receptor type 3; CXCR5, C–X–C chemokine receptor type 5; FNA, fine‐needle aspirate; FNB, fine‐needle biopsy; ICOS, inducible T‐cell costimulator; LN, lymph node; PD‐1, programmed cell death protein 1; T_mem_, T memory cell.

There has only been one FNA study focused on Tfh cells in healthy adults following SARS‐CoV‐2 vaccination (BNT162b2 mRNA).^57^ Another recently published study investigated the LN in healthy and SARS‐CoV‐2–infected individuals after SARS‐CoV‐2 vaccination, but used core biopsy samples to analyze the LN architecture.^58^ Mudd *et al*.^57^ sampled draining axillary LNs of SARS‐CoV‐2–naïve volunteers (*n* = 15) with longitudinal LN FNA samples taken before and after primary and secondary vaccination, and at increasing intervals up to 200 days after the first vaccination (*n* = 6).^57^ An HLA‐DPB1*04–restricted SARS‐CoV‐2 tetramer (S_167–180_) was used to detect S_167–180_‐specific Tfh cells present in the draining LN at day 40 after secondary vaccination in five individuals, and up to day 200 in two individuals (Figure 2). In peripheral blood the S_167–180_‐specific cTfh cells peaked 1 week after the second vaccination and then decreased thereafter (becoming undetectable at the later timepoints in some volunteers). TCR sequencing of the LN tetramer–specific Tfh cells was also performed in four participants at day 60, and in another three at day 110 (days 39 and 89 after secondary vaccination, respectively). Although specific dominant TCR clones remained consistent in the LN despite a 50‐day gap between sampling, a comparative analysis was not performed in the blood, which would have provided valuable insight into the clonal dynamics of both the memory CD4^+^ and the cTfh cells over time.

These studies emphasize a clear need for investigating T‐cell responses after vaccination at the site of induction across multiple different vaccines and cohorts. This could assist in settling the conflicting results in terms of the relationship between Tfh and cTfh cell lineages, but also further our understanding of the mechanisms of action by novel mRNA vaccines, which are now a highly attractive platform for immunization.

## RESPONSES TO SARS‐COV‐2 VACCINATION AMONG IMMUNOCOMPROMISED ADULTS

With increased diversity in SARS‐CoV‐2 variants and waning vaccination rates, immunocompromised patients are still at risk for more severe COVID‐19 symptoms and mortality.^59^ While antiviral treatments such as nirmatrelvir/ritonavir (Paxlovid) are effective in these groups, this protease inhibitor needs to be commenced within 5 days of symptom onset to have an optimal effect.^60^, ^61^ Monoclonal antibody therapy is also an important prevention modality to reduce mortality and disease burden; however, the rapid growth of new SARS‐CoV‐2 variants has reduced their efficacy and they are often expensive to manufacture and problematic to administer *en mass*.^62^ Vaccination therefore remains an important tool for reducing the effect of COVID‐19 with issues such as efficacy and appropriate seroconversion being key in these groups.

### Peripheral blood responses in immunocompromised individuals

There are limited studies focusing on the cTfh responses after vaccination in immunocompromised adults, therefore this section will include a number of studies describing vaccine‐specific CD4^+^ T cells and not particularly antigen‐specific cTfh in this population of interest.

In a recent study by Gao *et al*.^63^ spike‐specific CD4^+^ T‐cell responses were investigated in a number of immunocompromised patients, including those with solid organ transplants (*n* = 41), hematopoietic stem cell transplants (*n* = 43), chronic lymphocytic leukemia (*n* = 53), primary immunodeficiency (*n* = 48) and HIV infection (*n* = 50).^63^ The responses were compared across two BNT162b2 doses and followed out to 6 months. The patients with hematopoietic stem cell transplant, primary immunodeficiency and HIV had generally detectable and preserved spike‐specific CD4^+^ T‐cell responses, which have also been seen in both treatment‐naïve and immunochemotherapy‐treated individuals with lymphoma.^64^ Interestingly, only after the second vaccine dose was there a substantial and likely meaningful increase in spike‐specific CD4^+^ T cells recorded in the solid organ transplant and leukemia patients. At the 6‐month timepoint, the longevity of the CD4^+^ T‐cell responses in these latter groups (solid organ transplant and chronic lymphocytic leukemia) was not sustained as well as responses seen in healthy individuals or the other immunocompromised groups. While this study did not investigate the cTfh cells specifically, it does highlight meaningful differences between these patient groups with respect to the antigen‐specific CD4^+^ T‐cell responses, and this may flag which groups require more focus on to improve vaccine responses.

Apostolidis *et al*.^40^ recently analyzed cTfh cells from patients with multiple sclerosis treated with anti‐CD20 therapy (*n* = 20).^40^ In this patient group, the antigen‐specific cTfh cells, determined by SARS‐CoV‐2 peptide pool stimulation, had a nonsignificant reduction at all timepoints compared with healthy adults. Verstegen *et al*.^48^ have reported similar results when comparing patients with multiple sclerosis treated with anti‐CD20 (*n* = 7) and patients with rheumatoid arthritis on methotrexate (*n* = 14), both infected with SARS‐CoV‐2 prior to vaccination.^48^ They found a negligible or minimal increase in the cTfh and cTfh‐1 cell compartment (per μL of blood and as a percentage of memory CD4^+^ cells) following the first mRNA vaccination compared with a previously infected healthy control group. These findings could suggest that the recall of cTfh cells upon the reintroduction of SARS‐CoV‐2 could be impaired, although this finding could also be due to the lower cell numbers seen before vaccination compared with the healthy group in the latter paper.

All of these findings indicate potential deficiencies within the LN response, specifically as anti‐CD20 monoclonal antibody treatment reduces the number of B cells in the blood over time.^65^ The lack of interaction between LN Tfh and B cells could lead to reduced trafficking of the cTfh into the peripheral blood. Site‐specific analysis of secondary lymphoid organs would likely unveil possible issues with cell interactions within patients with multiple sclerosis treated with anti‐CD20. This also emphasizes the importance of undertaking thorough analyses of all subsets of memory CD4^+^ T cells when studying T‐cell responses and their possible functionality. As cTfh cells have been shown to positively correlate with NAb titers and are likely representative of Tfh cell action, this lack of investigation could potentially lead to an overestimation of effective T‐cell activity.

Overall, these studies emphasize the complexities across multiple immunocompromised patient groups. Differences in their specific T‐cell and antibody responses could potentially help explain the genesis of differences in vaccine efficacy. Despite the value of this work, it is clear that investigation needs to be performed in the site‐specific areas of the LN. As the cTfh cell compartment is reduced in those on anti‐CD20 therapy, the logical next step would be to perform similar LN studies in these patient groups to those performed in healthy volunteers. This would provide invaluable data on the interaction between B and T cells in the LN and likely inform the deficiencies within this area that could be manipulated to possibly increase vaccine efficacy.

### Lymph node responses in immunocompromised adults

As noted previously, there are limited studies that have investigated the LN immune response to vaccination, while these studies in immunocompromised adults are even rarer. Prior to the release of the SARS‐CoV‐2 vaccinations, there was only one such study investigating the LN responses in people living with HIV and on long‐term therapy. Moysi *et al*.^66^ performed core biopsies on the draining and nondraining inguinal LNs before and after influenza vaccination.^66^ Their study highlighted that there were cell expansions and gene expression differences in Tfh cells, after vaccination in people living with HIV and uninfected adults, despite long‐term therapy.

Furthermore, at time of writing, there was currently only one published study of LN FNBs on immunocompromised participants. Lederer *et al*.^67^ compared LN FNB samples from healthy participants (*n* = 15) and those on immunosuppressant drugs following kidney transplant (*n* = 13).^67^ Draining and nondraining (*n* = 4) LNs were sampled 14 days following primary and 8 days following secondary SARS‐CoV‐2 mRNA vaccination. In healthy individuals, similar to our previous LN study,^55^ they found that Tfh cell expansion was exclusively localized in the draining LN, and there was an increased percentage of these cells (from the total CD4^+^CD45RA^−^ memory T‐cell population) upon secondary vaccination. However, this was not the case for the kidney transplant group, which had a significant reduction in Tfh cells as the percentage of total CD4^+^ T cells in the LN. Clustering analysis of flow cytometry data using t‐distributed stochastic neighbor embedding of LN memory CXCR5^+^ CD4^+^ T cells revealed a clear reduction in the expression of PD‐1, Bcl‐6, ICOS and CD38 in the memory T cells from kidney transplant participants compared with healthy individuals. This emphasizes the marked failure of Tfh cell induction after vaccination in these immunocompromised individuals.

This decrease in Tfh cells was also reflected in the peripheral blood of the kidney transplant group, where peptide stimulation identified a reduction in the CD4^+^ T cells responding to SARS‐CoV‐2 across all vaccine timepoints in the immunocompromised group (including the third dose). These findings were similar to what was observed in the peripheral blood by Apostolidis *et al*.^40^ These and future studies could aid in the exploration of the effects of various adjuvants to supplement current vaccine formats aimed at improving efficacy or to enhance Tfh cell expansion.

LN follicle issues have also been observed in those undergoing antitumor necrosis factor‐α treatment for conditions such as inflammatory bowel disease.^68^ This type of immunosuppressive therapy decreases follicle numbers in the LN and leads to disrupted LN architecture as a whole. While this group mostly has an intact peripheral blood T‐cell response after SARS‐CoV‐2 vaccination, there could be underlying issues within the LN itself, making it an interesting area for FNB analysis.^68^, ^69^

Further research into SARS‐CoV‐2–specific germinal center Tfh cells and their implications on NAb levels is warranted. While investigating highly immunosuppressed patients such as organ transplant recipients provides valuable insight into improving severely depleted SARS‐CoV‐2 responses, there is a great need to improve vaccine efficacy in those that have autoimmune diseases or are on treatments such as anti‐CD20 monoclonal therapy. This would be assisted by investigating the immune responses at its source rather than repeated peripheral blood studies.

## CONCLUSION

It is clear that despite the large number of studies investigating peripheral blood spike–specific CD4^+^ and cTfh cells from healthy and immunocompromised adults after SARS‐CoV‐2 vaccination, there are areas that need further investigation. Standardization of the phenotypic markers used to define cTfh cells would be highly beneficial at identifying this CD4^+^ T‐cell subset, as several of the papers referenced in this review use CXCR5 exclusively, without incorporating PD‐1. Future clonality studies involving the LN would give more clarity to this standardization, as well as possibly incorporating markers such as CXCR3, ICOS, CD38 or HLA‐DR to identify acute specific cTfh cells after infection or vaccination. In addition, investigating approaches to increase vaccine efficacy in immunocompromised individuals would be highly beneficial. As these groups are often the most impacted by infections such as SARS‐CoV‐2, research to increase effective seroconversion would greatly reduce associated mortality and morbidity. Further research into vaccine efficacy would be greatly assisted by site‐specific LN analyses, which then has the potential to be modified to improve the immune response.

## AUTHOR CONTRIBUTIONS

**Mollie Ailie Acheson Boyd:** Writing – original draft; writing – review and editing. **Alexandra Carey Hoppe:** Writing – review and editing. **Anthony D Kelleher:** Supervision; writing – original draft; writing – review and editing. **C Mee Ling Munier:** Supervision; conceptualization; writing – original draft; writing – review and editing.

## CONFLICT OF INTEREST

The authors declare that they have no known competing financial interests or personal relationships that could have appeared to influence the work reported in this paper.

## References

[1] Doherty M , Buchy P , Standaert B , Giaquinto C , Prado‐Cohrs D . Vaccine impact: benefits for human health. Vaccine 2016; 34: 6707–6714. 27773475 10.1016/j.vaccine.2016.10.025

[2] Vilchez RA , McCurry K , Dauber J , *et al*. Influenza virus infection in adult solid organ transplant recipients. Am J Transplant 2002; 2: 287–291. 12096793 10.1034/j.1600-6143.2002.20315.x

[3] Couch RB , Englund JA , Whimbey E . Respiratory viral infections in immunocompetent and immunocompromised persons. Am J Med 1997; 102: 2–9. discussion 25–26. 10.1016/S0002-9343(97)00003-X PMC7124320 10868136

[4] Lee A , Wong SY , Chai LYA , *et al*. Efficacy of covid‐19 vaccines in immunocompromised patients: systematic review and meta‐analysis. BMJ 2022; 376: e068632. 35236664 10.1136/bmj-2021-068632 PMC8889026

[5] Beck CR , McKenzie BC , Hashim AB , *et al*. Influenza vaccination for immunocompromised patients: summary of a systematic review and meta‐analysis. Influenza Other Respi Viruses 2013; 7(Suppl 2): 72–75. 10.1111/irv.12084 PMC5909396 24034488

[6] Schramm R , Costard‐Jackle A , Rivinius R , *et al*. Poor humoral and T‐cell response to two‐dose SARS‐CoV‐2 messenger RNA vaccine BNT162b2 in cardiothoracic transplant recipients. Clin Res Cardiol 2021; 110: 1142–1149. 34241676 10.1007/s00392-021-01880-5 PMC8267767

[7] Westra J , van Assen S , Wilting KR , *et al*. Rituximab impairs immunoglobulin (Ig)M and IgG (subclass) responses after influenza vaccination in rheumatoid arthritis patients. Clin Exp Immunol 2014; 178: 40–47. 24889761 10.1111/cei.12390 PMC4360192

[8] Oyaert M , De Scheerder MA , Van Herrewege S , *et al*. Evaluation of humoral and cellular responses in SARS‐CoV‐2 mRNA vaccinated immunocompromised patients. Front Immunol 2022; 13: 858399. 35401575 10.3389/fimmu.2022.858399 PMC8988283

[9] Vinuesa CG , Linterman MA , Yu D , MacLennan IC . Follicular helper T cells. Annu Rev Immunol 2016; 34: 335–368. 26907215 10.1146/annurev-immunol-041015-055605

[10] Huber JE , Ahlfeld J , Scheck MK , *et al*. Dynamic changes in circulating T follicular helper cell composition predict neutralising antibody responses after yellow fever vaccination. Clin Transl Immunology 2020; 9: e1129. 32419947 10.1002/cti2.1129 PMC7221214

[11] Yin M , Xiong Y , Liang D , *et al*. Circulating Tfh cell and subsets distribution are associated with low‐responsiveness to hepatitis B vaccination. Mol Med 2021; 27: 32. 33794763 10.1186/s10020-021-00290-7 PMC8015036

[12] Herati RS , Reuter MA , Dolfi DV , *et al*. Circulating CXCR5^+^PD‐1^+^ response predicts influenza vaccine antibody responses in young adults but not elderly adults. J Immunol 2014; 193: 3528–3537. 25172499 10.4049/jimmunol.1302503 PMC4170011

[13] Krishnaswamy JK , Alsen S , Yrlid U , Eisenbarth SC , Williams A . Determination of T follicular helper cell fate by dendritic cells. Front Immunol 2018; 9: 2169. 30319629 10.3389/fimmu.2018.02169 PMC6170619

[14] Schmitt N , Morita R , Bourdery L , *et al*. Human dendritic cells induce the differentiation of interleukin‐21‐producing T follicular helper‐like cells through interleukin‐12. Immunity 2009; 31: 158–169. 19592276 10.1016/j.immuni.2009.04.016 PMC2731623

[15] Choi YS , Yang JA , Yusuf I , *et al*. Bcl6 expressing follicular helper CD4 T cells are fate committed early and have the capacity to form memory. J Immunol 2013; 190: 4014–4026. 23487426 10.4049/jimmunol.1202963 PMC3626566

[16] Choi YS , Kageyama R , Eto D , *et al*. ICOS receptor instructs T follicular helper cell versus effector cell differentiation via induction of the transcriptional repressor Bcl6. Immunity 2011; 34: 932–946. 21636296 10.1016/j.immuni.2011.03.023 PMC3124577

[17] Ma CS , Deenick EK , Batten M , Tangye SG . The origins, function, and regulation of T follicular helper cells. J Exp Med 2012; 209: 1241–1253. 22753927 10.1084/jem.20120994 PMC3405510

[18] Webb LMC , Linterman MA . Signals that drive T follicular helper cell formation. Immunology 2017; 152: 185–194. 28628194 10.1111/imm.12778 PMC5588773

[19] Deenick EK , Ma CS . The regulation and role of T follicular helper cells in immunity. Immunology 2011; 134: 361–367. 22043829 10.1111/j.1365-2567.2011.03487.x PMC3230790

[20] Rashu R , Bhuiyan TR , Hoq MR , *et al*. Cognate T and B cell interaction and association of follicular helper T cells with B cell responses in *Vibrio cholerae* O1 infected Bangladeshi adults. Microbes Infect 2019; 21: 176–183. 30580014 10.1016/j.micinf.2018.12.002 PMC6588510

[21] Stebegg M , Kumar SD , Silva‐Cayetano A , Fonseca VR , Linterman MA , Graca L . Regulation of the germinal center response. Front Immunol 2018; 9: 2469. 30410492 10.3389/fimmu.2018.02469 PMC6209676

[22] He J , Tsai LM , Leong YA , *et al*. Circulating precursor CCR7^lo^PD‐1^hi^ CXCR5^+^ CD4^+^ T cells indicate Tfh cell activity and promote antibody responses upon antigen reexposure. Immunity 2013; 39: 770–781. 24138884 10.1016/j.immuni.2013.09.007

[23] Brenna E , Davydov AN , Ladell K , *et al*. CD4^+^ T follicular helper cells in human tonsils and blood are clonally convergent but divergent from non‐Tfh CD4^+^ cells. Cell Rep 2020; 30: 137–152. e135. 31914381 10.1016/j.celrep.2019.12.016 PMC7029615

[24] Vella LA , Buggert M , Manne S , *et al*. T follicular helper cells in human efferent lymph retain lymphoid characteristics. J Clin Invest 2019; 129: 3185–3200. 31264971 10.1172/JCI125628 PMC6668682

[25] Bentebibel SE , Lopez S , Obermoser G , *et al*. Induction of ICOS^+^CXCR3^+^CXCR5^+^ TH cells correlates with antibody responses to influenza vaccination. Sci Transl Med 2013; 5: 176ra132. 10.1126/scitranslmed.3005191 PMC3621097 23486778

[26] Morita R , Schmitt N , Bentebibel SE , *et al*. Human blood CXCR5^+^CD4^+^ T cells are counterparts of T follicular cells and contain specific subsets that differentially support antibody secretion. Immunity 2011; 34: 108–121. 21215658 10.1016/j.immuni.2010.12.012 PMC3046815

[27] Wang Z , Wang Z , Diao Y , Qian X , Zhu N , Dong W . Circulating follicular helper T cells in Crohn's disease (CD) and CD‐associated colorectal cancer. Tumour Biol 2014; 35: 9355–9359. 24943684 10.1007/s13277-014-2208-2

[28] Chan JA , Loughland JR , de Labastida RF , *et al*. Th2‐like T follicular helper cells promote functional antibody production during plasmodium falciparum infection. Cell Rep Med 2020; 1: 100157. 33377128 10.1016/j.xcrm.2020.100157 PMC7762767

[29] Velu V , Mylvaganam GH , Gangadhara S , *et al*. Induction of Th1‐biased T follicular helper (Tfh) cells in lymphoid tissues during chronic simian immunodeficiency virus infection defines functionally distinct germinal center Tfh cells. J Immunol 2016; 197: 1832–1842. 27481845 10.4049/jimmunol.1600143 PMC4992610

[30] Velu V , Mylvaganam G , Ibegbu C , Amara RR . Tfh1 cells in germinal centers during chronic HIV/SIV infection. Front Immunol 2018; 9: 1272. 29928280 10.3389/fimmu.2018.01272 PMC5997779

[31] Hopkins J. COVID‐19 Dashboard. Johns Hopkins University Center for Systems Science and Engineering (CSSE) [updated 01/11/2022]. Available from: https://coronavirus.jhu.edu/map.html. 10.1016/S1473-3099(22)00434-0 PMC9432867 36057267

[32] Baden LR , El Sahly HM , Essink B , *et al*. Efficacy and safety of the mRNA‐1273 SARS‐CoV‐2 vaccine. N Engl J Med 2021; 384: 403–416. 33378609 10.1056/NEJMoa2035389 PMC7787219

[33] Polack FP , Thomas SJ , Kitchin N , *et al*. Safety and efficacy of the BNT162b2 mRNA Covid‐19 vaccine. N Engl J Med 2020; 383: 2603–2615. 33301246 10.1056/NEJMoa2034577 PMC7745181

[34] Voysey M , Clemens SAC , Madhi SA , *et al*. Safety and efficacy of the ChAdOx1 nCoV‐19 vaccine (AZD1222) against SARS‐CoV‐2: an interim analysis of four randomised controlled trials in Brazil, South Africa, and the UK. Lancet 2021; 397: 99–111. 33306989 10.1016/S0140-6736(20)32661-1 PMC7723445

[35] Sadoff J , Gray G , Vandebosch A , *et al*. Safety and efficacy of single‐dose Ad26.COV2.S vaccine against Covid‐19. N Engl J Med 2021; 384: 2187–2201. 33882225 10.1056/NEJMoa2101544 PMC8220996

[36] Zhang Y , Zeng G , Pan H , *et al*. Safety, tolerability, and immunogenicity of an inactivated SARS‐CoV‐2 vaccine in healthy adults aged 18‐59 years: a randomised, double‐blind, placebo‐controlled, phase 1/2 clinical trial. Lancet Infect Dis 2021; 21: 181–192. 33217362 10.1016/S1473-3099(20)30843-4 PMC7832443

[37] Logunov DY , Dolzhikova IV , Shcheblyakov DV , *et al*. Safety and efficacy of an rAd26 and rAd5 vector‐based heterologous prime‐boost COVID‐19 vaccine: an interim analysis of a randomised controlled phase 3 trial in Russia. Lancet 2021; 397: 671–681. 33545094 10.1016/S0140-6736(21)00234-8 PMC7852454

[38] Zhang Z , Mateus J , Coelho CH , *et al*. Humoral and cellular immune memory to four COVID‐19 vaccines. Cell 2022; 185: 2434–2451. 35764089 10.1016/j.cell.2022.05.022 PMC9135677

[39] Goel RR , Painter MM , Apostolidis SA , *et al*. mRNA vaccines induce durable immune memory to SARS‐CoV‐2 and variants of concern. Science 2021; 374: abm0829. 34648302 10.1126/science.abm0829 PMC9284784

[40] Apostolidis SA , Kakara M , Painter MM , *et al*. Cellular and humoral immune responses following SARS‐CoV‐2 mRNA vaccination in patients with multiple sclerosis on anti‐CD20 therapy. Nat Med 2021; 27: 1990–2001. 34522051 10.1038/s41591-021-01507-2 PMC8604727

[41] Spensieri F , Siena E , Borgogni E , *et al*. Early rise of blood T follicular helper cell subsets and baseline immunity as predictors of persisting late functional antibody responses to vaccination in humans. PLoS One 2016; 11: e0157066. 27336786 10.1371/journal.pone.0157066 PMC4918887

[42] Bentebibel SE , Khurana S , Schmitt N , *et al*. ICOS^+^PD‐1^+^CXCR3^+^ T follicular helper cells contribute to the generation of high‐avidity antibodies following influenza vaccination. Sci Rep 2016; 6: 26494. 27231124 10.1038/srep26494 PMC4882544

[43] Abudulai LN , Fernandez S , Corscadden K , *et al*. Production of IgG antibodies to pneumococcal polysaccharides is associated with expansion of ICOS^+^ circulating memory T follicular‐helper cells which is impaired by HIV infection. PLoS One 2017; 12: e0176641. 28463977 10.1371/journal.pone.0176641 PMC5413043

[44] Muselman ARSH , Vella L , Laura S , Tebas P , Wherry EJ . Identification of flu‐specific ICOS^+^CD38^+^ cTfh after influenza vaccination. J Immunol 2016; 196: 145.

[45] Wragg KM , Lee WS , Koutsakos M , *et al*. Establishment and recall of SARS‐CoV‐2 spike epitope‐specific CD4^+^ T cell memory. Nat Immunol 2022; 23: 768–780. 35314848 10.1038/s41590-022-01175-5

[46] Painter MM , Mathew D , Goel RR , *et al*. Rapid induction of antigen‐specific CD4^+^ T cells is associated with coordinated humoral and cellular immunity to SARS‐CoV‐2 mRNA vaccination. Immunity 2021; 54: e2133–e2142. e3. 10.1016/j.immuni.2021.08.001 PMC8361141 34453880

[47] Matsui K , Adelsberger JW , Kemp TJ , Baseler MW , Ledgerwood JE , Pinto LA . Circulating CXCR5^+^CD4^+^ T follicular‐like helper cell and memory B cell responses to human papillomavirus vaccines. PLoS One 2015; 10: e0137195. 26333070 10.1371/journal.pone.0137195 PMC4557948

[48] Verstegen NJM , Hagen RR , van den Dijssel J , *et al*. Immune dynamics in SARS‐CoV‐2 experienced immunosuppressed rheumatoid arthritis or multiple sclerosis patients vaccinated with mRNA‐1273. Elife 2022; 11: e77969. 35838348 10.7554/eLife.77969 PMC9337853

[49] Herati RS , Muselman A , Vella L , *et al*. Successive annual influenza vaccination induces a recurrent oligoclonotypic memory response in circulating T follicular helper cells. Sci Immunol 2017; 2: eaag2152. 28620653 10.1126/sciimmunol.aag2152 PMC5469419

[50] Samanovic MI , Cornelius AR , Gray‐Gaillard SL , *et al*. Robust immune responses are observed after one dose of BNT162b2 mRNA vaccine dose in SARS‐CoV‐2‐experienced individuals. Sci Transl Med 2022; 14: eabi8961. 34874183 10.1126/scitranslmed.abi8961 PMC9248013

[51] Chen Y , Chen L , Yin S , *et al*. The third dose of CoronVac vaccination induces broad and potent adaptive immune responses that recognize SARS‐CoV‐2 Delta and Omicron variants. Emerg Microbes Infect 2022; 11: 1524–1536. 35608053 10.1080/22221751.2022.2081614 PMC9176682

[52] Rydyznski Moderbacher C , Kim C , Mateus J , *et al*. NVX‐CoV2373 vaccination induces functional SARS‐CoV‐2‐specific CD4^+^ and CD8^+^ T cell responses. J Clin Invest 2022; 132: e160898. 35943810 10.1172/JCI160898 PMC9525112

[53] Gao X , Luo K , Wang D , *et al*. T follicular helper 17 (Tfh17) cells are superior for immunological memory maintenance. Elife 2023; 12: e82217. 36655976 10.7554/eLife.82217 PMC9891720

[54] Turner JS , Zhou JQ , Han J , *et al*. Human germinal centres engage memory and naive B cells after influenza vaccination. Nature 2020; 586: 127–132. 32866963 10.1038/s41586-020-2711-0 PMC7566073

[55] Law H , Mach M , Howe A , *et al*. Early expansion of CD38^+^ICOS^+^ GC Tfh in draining lymph nodes during influenza vaccination immune response. iScience 2022; 25: 103656. 35028536 10.1016/j.isci.2021.103656 PMC8741621

[56] Hey‐Nguyen WJ , Xu Y , Pearson CF , *et al*. Quantification of residual germinal center activity and HIV‐1 DNA and RNA levels using fine needle biopsies of lymph nodes during antiretroviral therapy. AIDS Res Hum Retroviruses 2017; 33: 648–657. 28287825 10.1089/aid.2016.0171

[57] Mudd PA , Minervina AA , Pogorelyy MV , *et al*. SARS‐CoV‐2 mRNA vaccination elicits a robust and persistent T follicular helper cell response in humans. Cell 2022; 185: 603–613. e615. 35026152 10.1016/j.cell.2021.12.026 PMC8695127

[58] Roltgen K , Nielsen SCA , Silva O , *et al*. Immune imprinting, breadth of variant recognition, and germinal center response in human SARS‐CoV‐2 infection and vaccination. Cell 2022; 185: 1025–1040. e1014. 35148837 10.1016/j.cell.2022.01.018 PMC8786601

[59] Fung M , Babik JM . COVID‐19 in immunocompromised hosts: what we know so far. Clin Infect Dis 2021; 72: 340–350. 33501974 10.1093/cid/ciaa863 PMC7337668

[60] Hedvat J , Lange NW , Salerno DM , *et al*. COVID‐19 therapeutics and outcomes among solid organ transplant recipients during the omicron BA.1 era. Am J Transplant 2022; 22: 2682–2688. 35801839 10.1111/ajt.17140 PMC9349644

[61] Wen W , Chen C , Tang J , *et al*. Efficacy and safety of three new oral antiviral treatment (molnupiravir, fluvoxamine and Paxlovid) for COVID‐19: a meta‐analysis. Ann Med 2022; 54: 516–523. 35118917 10.1080/07853890.2022.2034936 PMC8820829

[62] Focosi D , McConnell S , Casadevall A , Cappello E , Valdiserra G , Tuccori M . Monoclonal antibody therapies against SARS‐CoV‐2. Lancet Infect Dis 2022; 22: e311–e326. 35803289 10.1016/S1473-3099(22)00311-5 PMC9255948

[63] Gao Y , Cai C , Wullimann D , *et al*. Immunodeficiency syndromes differentially impact the functional profile of SARS‐CoV‐2‐specific T cells elicited by mRNA vaccination. Immunity 2022; 55: 1732–1746. e1735. 35961317 10.1016/j.immuni.2022.07.005 PMC9293955

[64] Beaton B , Sasson SC , Rankin K , *et al*. Patients with treated indolent lymphomas immunized with BNT162b2 have reduced anti‐spike neutralizing IgG to SARS‐CoV‐2 variants, but preserved antigen‐specific T cell responses. Am J Hematol 2023; 98: 131–139. 35607995 10.1002/ajh.26619 PMC9349368

[65] van Lierop ZY , Wieske L , Koel‐Simmelink MJ , *et al*. Serum contactin‐1 as a biomarker of long‐term disease progression in natalizumab‐treated multiple sclerosis. Mult Scler 2022; 28: 102–110. 33890520 10.1177/13524585211010097 PMC8689420

[66] Moysi E , Pallikkuth S , De Armas LR , *et al*. Altered immune cell follicular dynamics in HIV infection following influenza vaccination. J Clin Invest 2018; 128: 3171–3185. 29911996 10.1172/JCI99884 PMC6025971

[67] Lederer K , Bettini E , Parvathaneni K , *et al*. Germinal center responses to SARS‐CoV‐2 mRNA vaccines in healthy and immunocompromised individuals. Cell 2022; 185: 1008–1024. e15. 35202565 10.1016/j.cell.2022.01.027 PMC8808747

[68] Duncan VE , Chisholm KM , Pacheco MC . Effects of tumor necrosis factor α inhibitors on lymph node and ileocecal mucosa‐associated lymphoid tissue architecture in patients with inflammatory bowel disease. Pediatr Dev Pathol 2020; 23: 115–120. 31362580 10.1177/1093526619866371

[69] Alexander JL , Liu Z , Munoz Sandoval D , *et al*. COVID‐19 vaccine‐induced antibody and T‐cell responses in immunosuppressed patients with inflammatory bowel disease after the third vaccine dose (VIP): a multicentre, prospective, case‐control study. Lancet Gastroenterol Hepatol 2022; 7: 1005–1015. 36088954 10.1016/S2468-1253(22)00274-6 PMC9458592

